# Age-Dependent Corpus Callosum Thickness Abnormalities and Clinical Implications in Treatment-Naïve First-Episode Schizophrenia

**DOI:** 10.31083/AP48363

**Published:** 2026-04-20

**Authors:** Jinni Lin, Lihong Deng, Mingli Li, Qiang Wang, Liansheng Zhao, Hua Yu, Xiaojing Li, Wei Deng, Wanjun Guo, Tao Li, Peiyan Ni, Wei Wei

**Affiliations:** ^1^Affiliated Mental Health Center & Hangzhou Seventh People's Hospital, Zhejiang University School of Medicine, 310013 Hangzhou, Zhejiang, China; ^2^Nanhu Brain-computer Interface Institute, 311100 Hangzhou, Zhejiang, China; ^3^Mental Health Center of West China Hospital, Sichuan University, 610041 Chengdu, Sichuan, China; ^4^NHC and CAMS Key Laboratory of Medical Neurobiology, Zhejiang University, 310058 Hangzhou, Zhejiang, China; ^5^Liangzhu Laboratory, MOE Frontier Science Center for Brain Science and Brain-machine Integration, State Key Laboratory of Brain-machine Intelligence, Zhejiang University, 311121 Hangzhou, Zhejiang, China; ^6^Zhejiang Key Laboratory of Clinical and Basic Research for Psychiatric Diseases, 310013 Hangzhou, Zhejiang, China

**Keywords:** corpus callosum, magnetic resonance imaging, schizophrenia, white matter

## Abstract

**Background:**

Although morphological abnormalities of the corpus callosum (CC) have been reported in schizophrenia, findings across studies have been inconsistent. We systematically examined whether these morphological alterations are influenced by age.

**Methods:**

A total of 151 individuals with treatment-naïve first-episode schizophrenia (FES) and 278 healthy controls were included. T1-weighted structural MRI scans were used to segment the CC on the midsagittal plane into 100 equidistant points, and CC thickness was estimated at each point. To determine whether CC thickness abnormalities associated with schizophrenia were moderated by age, we applied the Johnson–Neyman technique. Additionally, we investigated the relationship between age-dependent CC thickness abnormalities and clinical symptoms using partial least-squares correlation analysis.

**Results:**

Abnormal CC thickness was observed in individuals with treatment-naïve FES, specifically within the rostral body, anterior midbody, isthmus, and splenium. These regions were thinner in younger patients compared with healthy controls but appeared thicker in older patients. Furthermore, increased CC thickness in older patients was associated with greater clinical symptom severity, whereas this association was not observed in younger patients.

**Conclusions:**

Our findings demonstrate that CC thickness abnormalities in treatment-naïve FES are age-dependent. The relationship between CC thickness and symptom severity also varies as a function of age. These results suggest that the CC may represent a critical biological target for age-sensitive, individualized therapeutic interventions in schizophrenia.

## Main Points

1. Corpus callosum (CC) thickness abnormalities in treatment-naïve first-episode schizophrenia (FES) were age-dependent: thinner in younger patients and thicker in older patients, compared with healthy controls.

2. Specific CC subregions driving these effects were the rostral body, anterior midbody, isthmus, and splenium.

3. Greater CC thickness in older FES patients was associated with more severe symptoms, a correlation not observed in younger patients.

4. The results suggest that adolescent-onset schizophrenia might represent a distinct form of the illness and implicate the CC as a potential target for age-sensitive, individualized therapeutic strategies.

## 1. Introduction

Schizophrenia (SZ) is a persistent and severe mental disorder characterized primarily by paranoid delusions and auditory hallucinations [1]. The societal and economic burden caused by SZ is substantial [2, 3]. Previous studies have found significant white matter anomalies in SZ patients. A meta-analysis of the enhancing neuroimaging genetics through meta-analysis consortium (ENIGMA) SZ diffusion tensor imaging (DTI) Working Group’s study, which involved over 4300 participants from 29 independent cohorts, revealed significant fractional anisotropy decreases in 20 of 25 major white matter regions of interest in individuals with SZ. Among these, the most pronounced effects were localized to the corpus callosum (CC) [4]. Furthermore, a meta-analysis conducted by Zhuo *et al*. [5] reported white-matter diffusion alterations in the CC of SZ patients, and identified the genu and splenium of the CC as two areas with a lower fractional anisotropy value than that of healthy controls (HCs).

The CC stands as the brain’s largest white matter structure, functioning as the primary commissural fiber bundle that facilitates communication between the two cerebral hemispheres [6]. Its morphological abnormalities in SZ are well-established, with numerous studies reporting a smaller area and volume. Two meta-analyses indicated a significantly smaller CC area among SZ patients, particularly in first-episode cases, than in HCs [7, 8]. Collinson *et al*. [9] found that SZ patients presented with substantially smaller CC areas as well as CC volumes than the control group. Nevertheless, the findings were inconsistent. Several studies also reported no significant differences in CC morphology between SZ and HCs [10, 11], or even larger CC areas in SZ [12].

This inconsistency may be influenced by sample-selection bias, such as variations in the age of onset of SZ patients across studies. Although SZ commonly emerges in early adulthood, a smaller group (5–18%) experiences their initial psychotic episode in childhood or adolescence; this is referred to as early-onset SZ (before age 18). Evidence has indicated that early-onset SZ often shows more severe symptoms and a poorer prognosis than does adult-onset SZ (after age 18) [13, 14, 15].

Although various methods exist for characterizing CC morphology, such as areal subdivision [16], cortical endpoint [17], and boundary tangent [18], the present study used the cross-sectional thickness model [19]. This model, which is a state-of-the-art approach, fills a significant methodological gap by providing higher regional specificity than do the other techniques. By generating 100 data points along the CC, it offers a considerable improvement in granularity over the five-to-seven subregions commonly derived from segment-specific methods. This allows for more precise insights and could offer improved sensitivity in detecting meaningful effects.

The primary purpose of this study was to investigate age-dependent CC-thickness abnormalities in first-episode schizophrenia (FES). Using a moderation analysis, we explored whether these abnormalities varied across different ages of onset. We also examine the association between identified CC thickness abnormalities and the clinical symptomatology of FES.

## 2. Materials and Methods

### 2.1 Participants

FES patients were recruited from both inpatient and outpatient psychiatric units at West China Hospital, Sichuan University. All patients were treatment-naïve. The diagnosis of SZ was determined by the criteria outlined in the Structured Clinical Interview for Mental Disorders, Fourth Edition (DSM-IV), patient version (SCID-P). HCs were recruited through community advertisements. Interested individuals from the community were invited to West China Hospital for screening and assessment using the SCID for DSM-IV, non-patient edition to ensure they had no current or past psychiatric disorders. General exclusion criteria for all participants included: individuals with severe physical diseases, nervous system diseases, personality disorders, alcohol abuse, substance abuse, or an intelligence quotient (IQ) below 70. Participants provided informed consent after receiving a full description of the study. For juveniles, the informed consent forms were signed by their legal guardians. This study was approved by the Institutional Review Board of West China Hospital of Sichuan University (2017-131) and was conducted in accordance with the Helsinki Declaration.

All study participants were of Han Chinese ethnicity and predominantly right-handed, as determined by the Annett Handedness Scale [20]. The Positive and Negative Syndrome Scale (PANSS) was estimated when patients were recruited. We used a four-factor model to categorize symptoms as estimated by PANSS, which included negative, positive, affective, and cognitive dimensions [21].

### 2.2 Image Acquisition

All neuroimaging scans were conducted using a 3.0 T MR scanner (Achieva; Philips, Amsterdam, Netherlands), equipped with an 8-channel phased-array head coil. Foam padding and earplugs were used to control participant head movement and suppress scanner noise.

High-resolution T1-weighted (T1w) images were acquired using a magnetization-prepared rapid gradient-echo sequence (MPRAGE) with the following parameters: repetition time (TR): 8.1 ms, echo time (TE): 3.7 ms, inversion time (TI): 1072.4 ms, flip angle: 7°, slice thickness: 1 mm (without slice gap), 188 axial slices, matrix size: 256 × 256, field of view (FOV): 256 × 256 mm, voxel size: 1 × 1 × 1 mm^3^. To ensure data quality, a rigorous quality control procedure was implemented for the assessment of T1w images (https://www.humanconnectome.org/storage/app/media/documentation/s1200/HCP_S1200_Release_Appendix_IV.pdf). Only images categorized as “good” or “excellent” were used in subsequent data processing and analysis; any scans with artifacts were repeated.

### 2.3 Thickness of the Corpus Callosum

**Fig. 1.**
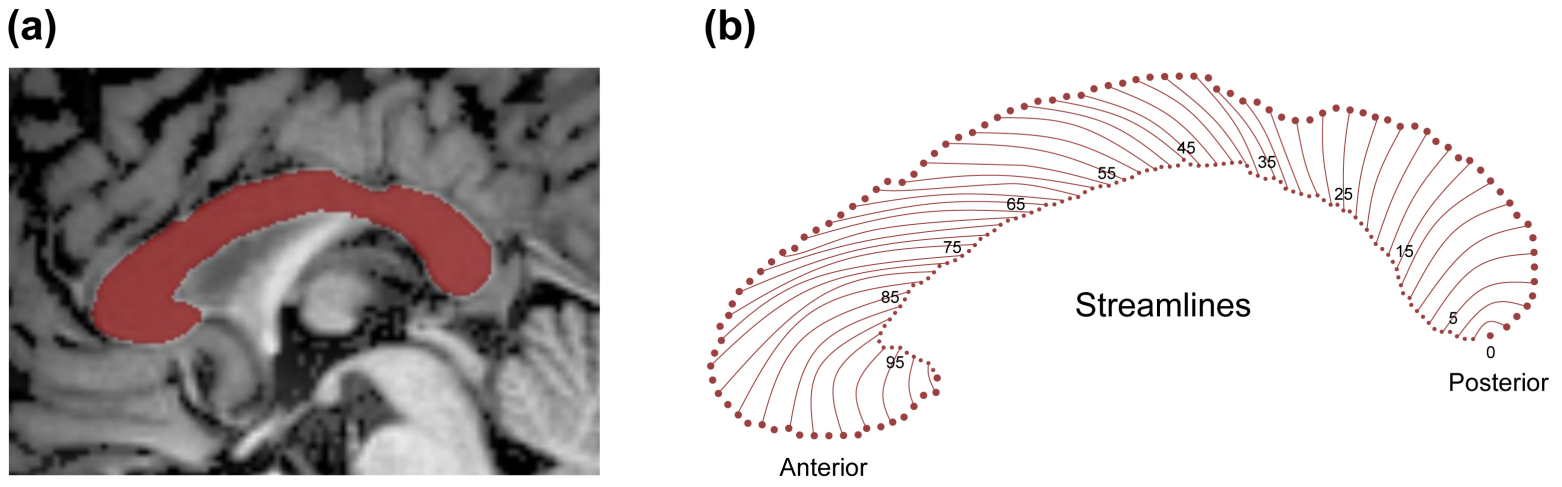
**Generation of CC thickness streamlines**. (a) Mid-sagittal CC segmentation in a sample subject, with the final CC mask shown in red. (b) Nonoverlapping cross-sectional contour lengths (streamlines) are indexed starting from 0 at the posterior junction of the inferior and superior boundaries of the CC, progressing anteriorly to node 100 at the most rostral point. CC, corpus callosum.

We estimated the thickness of the CC by using a semi-automated pipeline (https://github.com/chrisadamsonmcri/CCSegThickness), as comprehensively detailed in previous publications [19, 22]. This pipeline comprised several crucial steps: initial identification of the mid-sagittal plane from T1-weighted MRI data, followed by template-based segmentation of the CC, correction of topological errors, and exclusion of pericallosal blood vessels. Post-segmentation, thickness profiles were generated through the following procedures: the CC was partitioned into left and right halves, delimited by the bottommost extremities at the left and right. An intermediate equipotential contour was generated between these endpoints, computed as a solution to Laplace’s equation to optimize the length of the CC midline contour. This process also divided the outer boundary of the structure into superior and inferior contours. Streamlines were subsequently computed at regular intervals along the central contour, defined as non-overlapping parallel lines intersecting superior and inferior contours orthogonally in an anterior-posterior trajectory. For this study, we created 100 streamlines per subject (see generation of CC thickness streamlines in Fig. 1).

### 2.4 Statistical Analyses

#### 2.4.1 Demographic and Clinical Data 

We used chi-square tests or 2-sample *t*-tests to examine sex, age (range: 11–42 years), years of education (EDUY), and total intracranial volume (TIV) between groups. All statistical analyses were performed using IBM SPSS Statistics for Macintosh (IBM Corporation, Somers, NY, USA, Version 26.0).

#### 2.4.2 Primary Analysis: Group Differences in CC Thickness

We used the Johnson-Neyman (JN) technique [23, 24] with the “interactions” (v1.1.5) R package in R v 4.2.3 (R Foundation for Statistical Computing, Vienna, Austria) to explore the conditional effects (θ) of group (X) on CC thickness (Y) across the entire age range (11–42 years old), adjusting for sex, EDUY, and TIV as nuisance covariates. Traditional moderation analysis typically used the pick-a-point method, testing the conditional effects of X on Y at selected points of the continuous moderator (M). In contrast, the JN technique provided a comprehensive analysis by evaluating these effects throughout the entire range of M [25]. This allowed for the identification of specific ages at which the effect of X on Y becomes statistically significant, thereby avoiding the subjectivity inherent in traditional pick-a-point methods. Significance for multiple comparisons was adjusted using the False Discovery Rate (FDR) method, and the significance level was set as FDR-*p* ≤ 0.05.

#### 2.4.3 Exploratory Analysis: Relationship Between CC Thickness and Clinical Symptoms

To investigate whether the interaction between CC thickness and age was associated with clinical symptoms, we performed a Partial Least Squares Correlation (PLSC) analysis on the FES group [26, 27]. PLSC is a multivariate statistical method designed to identify latent variables (LVs) that maximize the covariance between two data matrices. Initially, we performed PLSC with the R matrix as a product of the design matrix (X) and the scores of PANSS (Y). The design matrix included CC thickness data, age, and their interaction. Subsequently, singular value decomposition was used to decompose matrix R, yielding pairs of saliences (U and V) along with their respective singular values. Finally, we computed participant-specific behavior scores by projecting individual PANSS scores onto respective saliences using the formula: behavior scores = YU. These behavior scores represented each participant’s symptom pattern captured by the LVs.

To determine statistical significance, a permutation test (10,000 iterations) was run to generate null distributions for all singular values collectively, setting the significance threshold at *p* ≤ 0.05. The Bootstrap ratio (BSR) was computed by dividing observed salience by the standard error (estimated from 10,000 samples). A BSR with a value higher than 2 or lower than –2 was deemed statistically significant (*p* ≤ 0.05) [26]. This method mitigated the issue of inflated degrees of freedom associated with multiple testing. Given the collective assessment of latent components via permutation testing and individual elements via bootstrapping, correction for multiple comparisons within a single PLSC analysis was unnecessary. In this study, PLSC analyses were performed separately for four subregions, and FDR correction was applied across these subregions to adjust for multiple comparisons. JN analysis between the behavior scores and CC thickness with age as a moderator was conducted to further confirm the relationship between CC thickness and clinical symptoms.

To account for potential confounding by clinical characteristics, we performed a sensitivity analysis. This analysis assessed the relationship between CC thickness (in regions identified as statistically significant in the primary analysis) and clinical symptoms using the JN technique, adjusting for illness duration, age of onset, sex, and years of education.

## 3. Results

### 3.1 Demographic and Clinical Data

**Fig. 2.**
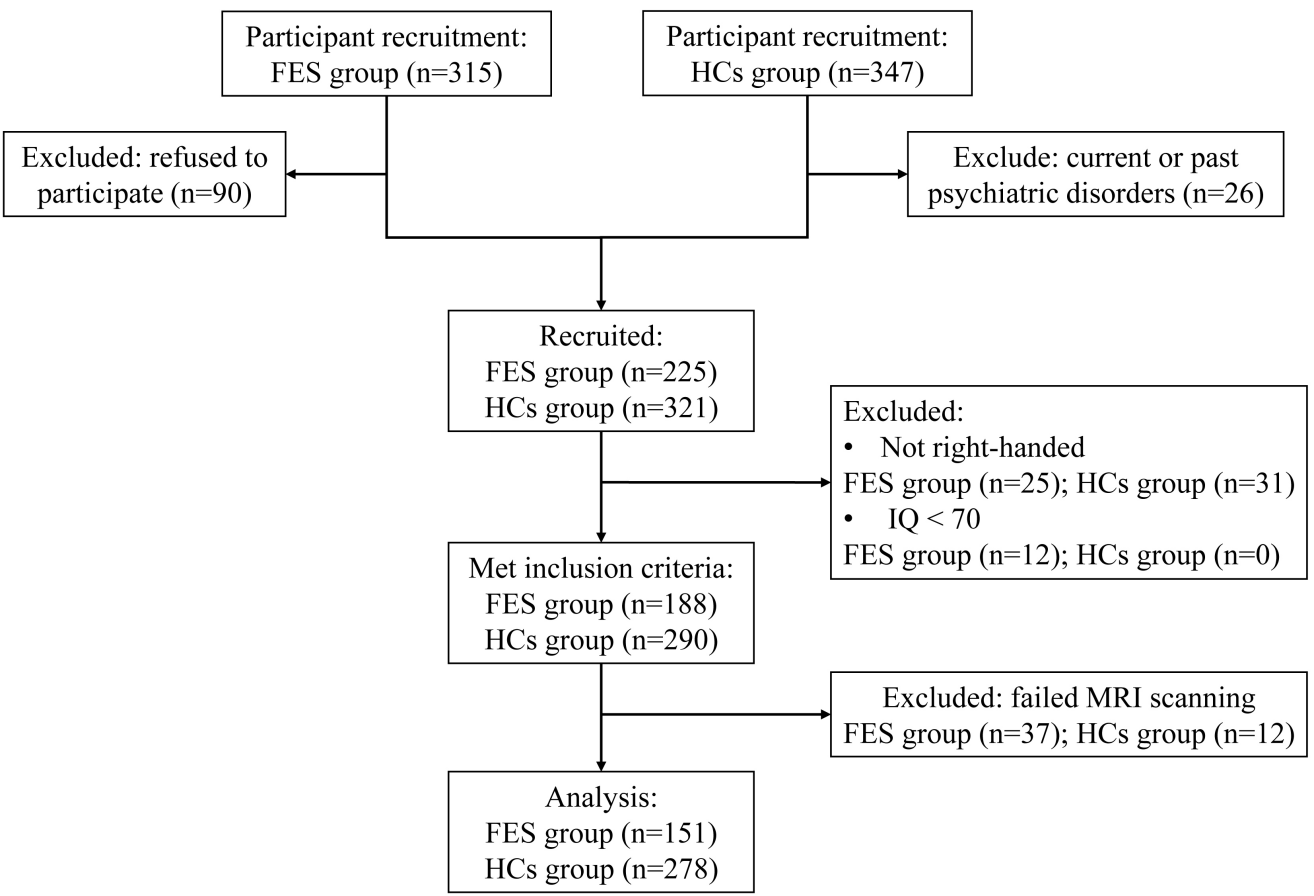
**Participant flow diagram**. Abbreviations: FES, first-episode schizophrenia; HCs, healthy controls.

Fig. 2 shows participant flow. The final sample comprised 151 FES patients and 278 HCs. Sex and TIV did not differ between FES patients and HCs. FES patients had significantly lower age and EDUY (both *p* < 0.001). Table 1 shows these results along with the PANSS scores of FES patients.

**Table 1.** **Demographic and clinical data**.

	FES (*n* = 151)	HC (*n* = 278)	Statistics	*p*
Age (years)	20.34 ± 6.93 (12.00–42.00)	23.71 ± 5.95 (11.00–42.00)	t = –5.06	<0.001
Gender (M/F)	59/92	92/186	χ^2^ = 1.534	0.216
EDUY (years)	11.27 ± 2.81 (6.00–18.00)	15.09 ± 3.17 (6.00–24.00)	t = –12.39	<0.001
TIV (cm^3^)	1449.59 ± 127.23 (1101.60–1821.62)	1446.47 ± 123.79 (1130.99–1862.85)	t = 0.25	0.805
PANSS	n = 137			
Total	83.91 ± 22.03 (32.00–143.00)			
Negative	8.22 ± 2.96 (2.54–16.55)			
Positive	7.59 ± 2.29 (2.44–12.29)			
Affective	7.00 ± 2.33 (2.66–12.84)			
Cognitive	8.26 ± 2.87 (2.96–15.77)			

Abbreviations: EDUY, years of education; TIV, total intracranial volume; PANSS, Positive and Negative Syndrome Scale. PANSS data were unavailable for 14 patients due to missing source records. Note: values are presented as mean ± standard deviation (minimum–maximum).

### 3.2 Primary Findings: Group Differences in CC Thickness

**Fig. 3.**
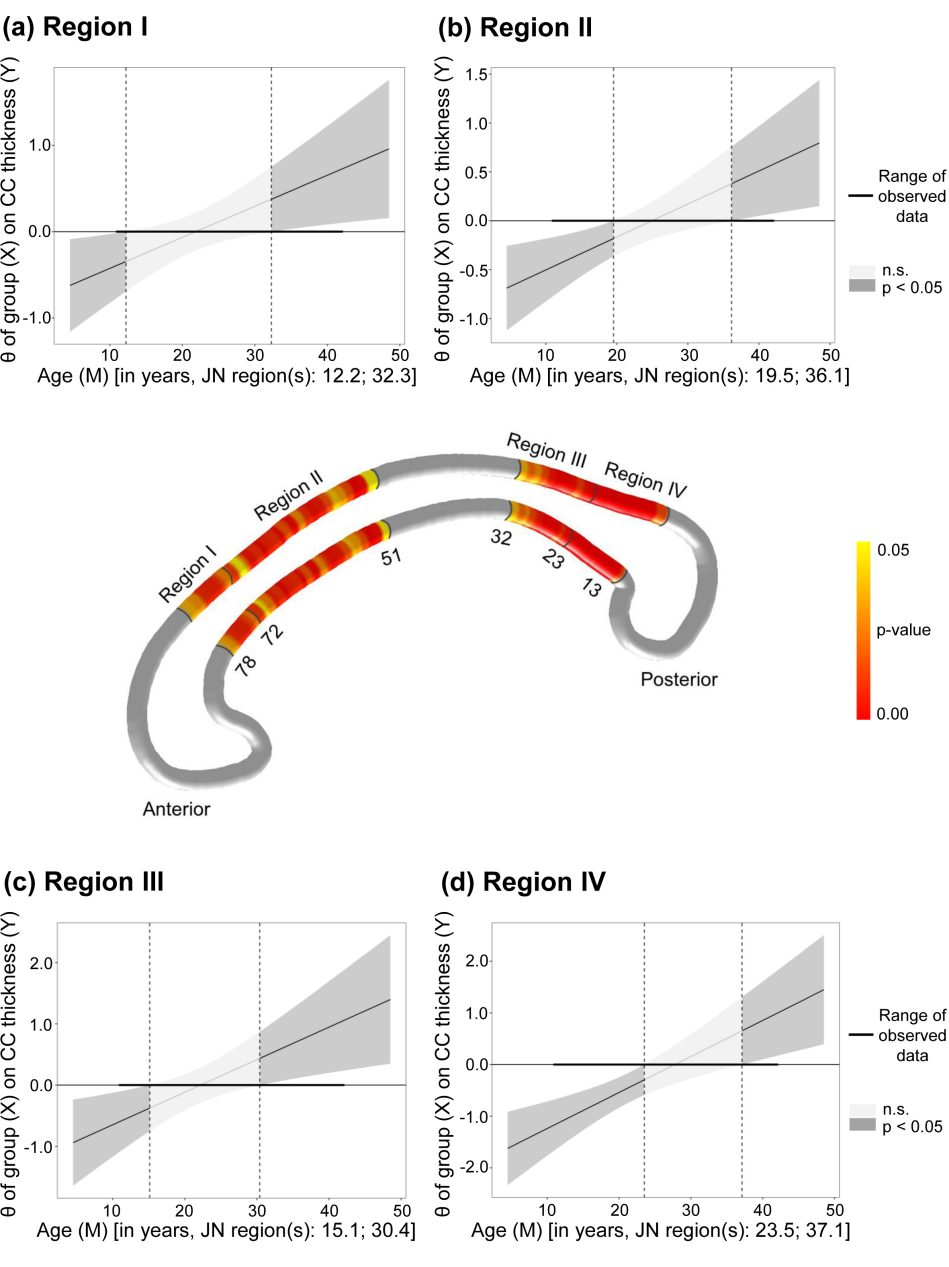
**Age-related group differences in CC thickness**. The middle panel illustrates significant *p*-values (*p* ≤ 0.05) for group × age interactions, displayed in light yellow on the CC surface model, with lower values shown in red. (a–d) Present the conditional effects (θ) of group on CC thickness across the entire age range using the Johnson-Neyman technique for Regions I through IV, respectively. M, moderator; JN, Johnson-Neyman; n.s., not statistically significant.

Preliminary JN analysis identified significant differences in CC thickness between FES patients and HCs, with the affected streamlines presented in Fig. 3. To facilitate a more focused and systematic analysis, we subsequently combined the results with Witelson’s widely accepted anatomical segmentation method [16], aggregating these streamlines of significant difference into four principal regions: Region I consisted of streamlines 73 to 78, corresponding to the anterior half of Witelson’s region 3 (rostral body); Region II consisted of streamlines 51 to 72, corresponding to Witelson’s region 4 (anterior midbody) and the posterior half of region 3; Region III consisted of streamlines 24 to 32, corresponding to Witelson’s region 6 (isthmus); and Region IV consisted of streamlines 13 to 23, corresponding to Witelson’s region 7 (splenium). The average thickness was computed for each of these four regions and subjected to further JN analysis.

Further JN analysis revealed that FES patients under 12.2, 19.5, 15.1, and 23.5 years of age exhibited thinner CC than did HCs in Regions Ⅰ to Ⅳ, respectively. Conversely, those over 32.3, 36.1, 30.4, and 37.1 years showed thicker CC than HCs in the same respective regions. The above results are presented in Fig. 3.

### 3.3 Exploratory Findings: Relationship Between CC Thickness and Clinical Symptoms

**Fig. 4.**
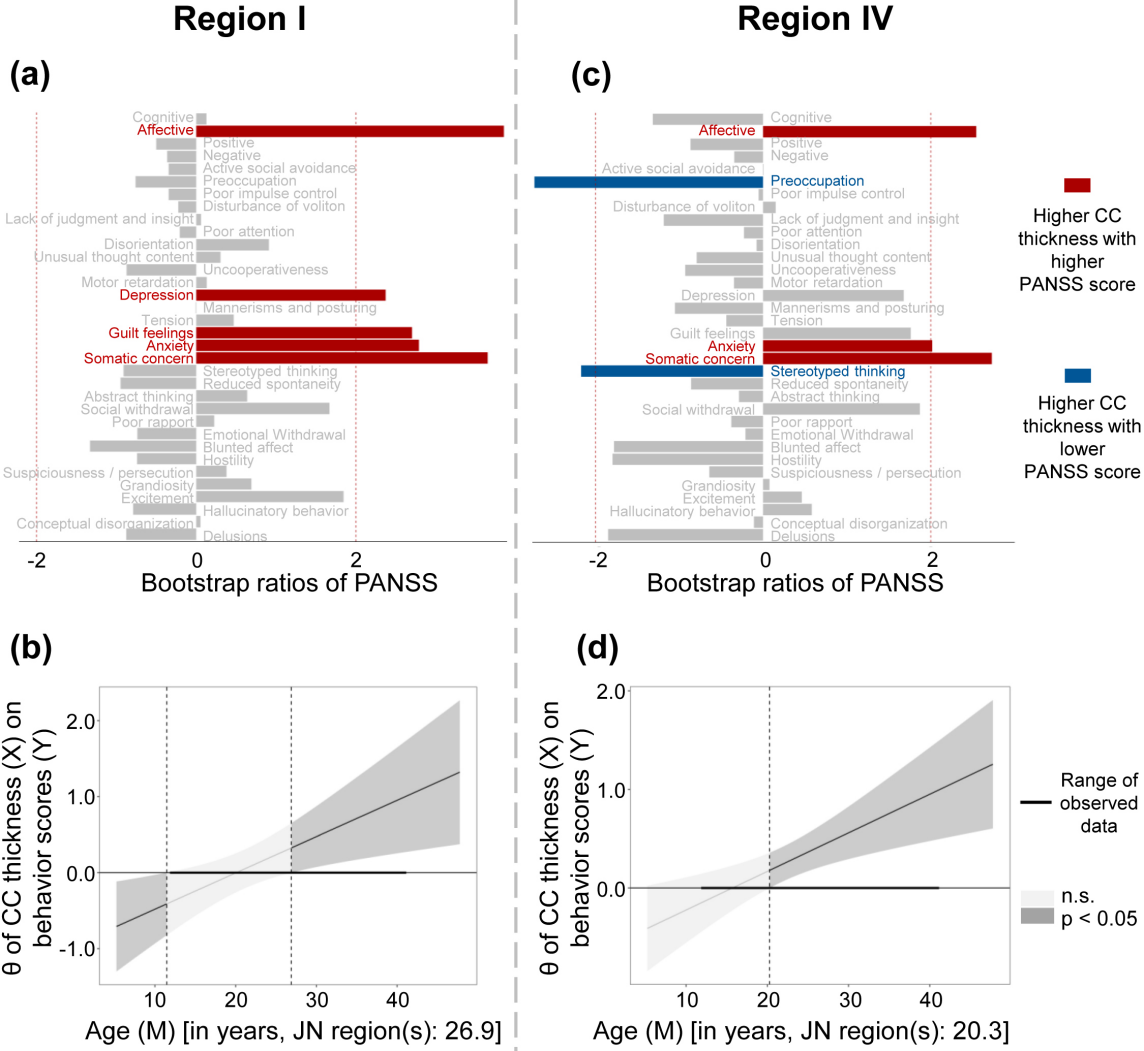
**Relationship between CC thickness and clinical symptoms**. (a,c) Symptoms shown in red were positively correlated with increased CC thickness, and symptoms shown in blue were negatively correlated with increased CC thickness. (b,d) The conditional effects (θ) of CC thickness on the behavior scores across the entire age range using the Johnson-Neyman technique. n.s., not statistically significant.

PLSC and subsequent JN analysis revealed that in Region Ⅰ, increased CC thickness was associated with higher G1 (somatic concern), G2 (anxiety), G3 (guilt feelings), G6 (depression), and affective-factor scores in FES patients older than 26.9 years old (all BSR >2, *p* < 0.05) (Fig. 4a,b). In Region Ⅱ, although PLSC analysis resulted in one significant LV (FDR-*p* = 0.045), the JN analysis showed that this interaction was not significant (*p* = 0.129). In Region Ⅲ, no significant LV was identified (FDR-*p* = 0.138). In Region Ⅳ, PLSC and JN analysis suggested that increased CC thickness was associated with higher G1 (somatic concern), G2 (anxiety), and affective-factor scores, and lower N7 (stereotyped thinking) and G15 (preoccupation) scores in FES patients over 20.3 years old (all BSR >2 or <–2, *p* < 0.05) (Fig. 4c,d).

After controlling for illness duration, age of onset, sex, and years of education as covariates, JN analysis revealed that in Region Ⅰ, greater CC thickness was significantly associated with greater symptom severity in FES patients older than 27.16 years, whereas less CC thickness was linked to more severe symptoms in those younger than 13.36 years. In Region Ⅳ, greater CC thickness was associated with more severe affective symptoms in patients older than 19.59 years.

## 4. Discussion

In this study, we used a well-validated mesh-modeling technique capturing CC thickness with extremely high regional specificity to investigate the CC abnormalities of treatment-naïve FES patients across a wide age range. The CC is generally thinner in younger patients (below cutoffs ranging from <12.2 to <23.5 years) than in HCs, and thicker in older ones (above cutoffs ranging from >30.4 to >37.1 years). In older FES patients, there exists a positive correlation between CC thickness and the severity of clinical symptoms.

Previous neuroimaging studies investigating CC morphology in SZ have yielded inconsistent findings, with some reporting CC thinning [28, 29] and others reporting CC thickening [30] or larger CC areas [12] in patients than in HCs. This apparent contradiction in the literature may largely stem from methodological differences, particularly concerning the age range of the participants. Studies reporting lower CC thickness predominantly enrolled relatively younger patient cohorts, whereas those reporting greater thickness tended to include older patients. Our study, by encompassing a broad age spectrum of FES patients, suggested that CC pathology in FES is fundamentally age-dependent.

Previous research has consistently demonstrated that the CC is smaller in younger FES patients than in HCs. As early as 2002, Keshavan *et al*. [31] reported reduced total CC area and smaller areas in several CC subregions in FES patients with an average age of 24.20 years. More recently, Huang *et al*. [32] similarly observed a significantly smaller whole CC area in a cohort of young FES patients (average age 22.40 years). Regarding CC thickness, Walterfang *et al*. [28] conducted a study involving 30 FES patients with an average age of 21.56 years, and found that CC thickness in FES was less than that of HCs. Furthermore, in a study of 160 FES patients (average age 24.21 years), Tao *et al*. [29] also reported less CC thickness in the posterior midbody subregion than in HCs. The findings of the present study aligned well with these previous investigations, further localizing less CC thickness in younger FES patients to four specific subregions: the rostral body, anterior midbody, isthmus, and splenium.

The maturation of the CC is known to continue throughout childhood and adolescence [33, 34]. This developmental context is crucial, as previous research has documented age-related increases in CC thickness, particularly within the posterior midbody and isthmus, during this period [6]. Our finding of a significantly thinner CC in younger FES patients may reflect a deviation from typical developmental patterns observed in HCs [35]. A longitudinal study of adolescent SZ patients and HCs, which reported delayed and altered maturation in various white matter tracts including the CC, provides more direct evidence supporting this notion [36].

In the current study, greater CC thickness was observed in older FES patients. This finding also aligned with previous research, dating back to Rosenthal and Bigelow’s 1972 report [37] of greater CC thickness in post-mortem brains of SZ patients. That observation was later replicated by Nasrallah *et al*. [30] using MRI. Furthermore, John *et al*. [12] observed larger areas in several CC subregions in FES patients (mean age 30.13 years) than in HCs. Similarly, Narr *et al*. [38] found greater CC width in patients (mean age 31.1 years) than in HCs. These previous observations were therefore corroborated again by our present findings.

A greater thickness of the CC observed on structural MRI may reflect a greater number of axons [39], suggesting enhanced structural connectivity between cerebral hemispheres. This heightened interhemispheric connectivity could potentially underlie the “abnormal functional hyperconnection” reported in SZ by David [40]. Beyond axonal proliferation, greater CC thickness may also result from various microstructural alterations, including gliosis, edema, abnormal myelination, or aberrant fiber organization. Indeed, previous investigations in SZ have reported more severe gliosis [41], extra-axonal edema [42], and dysregulated myelination [43] within the CC. Therefore, further research is necessary to precisely determine the specific pathophysiological mechanisms responsible for the observed structural difference in our study and to elucidate its functional implications.

Abnormalities in CC thickness, as identified in our research, were primarily localized to the rostral body, anterior midbody, isthmus, and splenium, regardless of whether the patients were younger or older. Substantial evidence from both monkeys and humans showed that the premotor, supplementary motor, and motor fibers typically traverse the CC rostral body and anterior midbody subregions, whereas the isthmus connects the parietal and superior temporal cortices, and the splenium connects the occipital, inferior temporal, and parietal regions [16, 17, 44, 45, 46, 47].

Previous research has consistently documented abnormalities in the rostral and anterior midbody subregions of the CC in individuals with SZ [48]. Alterations in motor tracts within these specific CC subregions have been proposed as a potential neurobiological substrate for catatonia in SZ [49, 50]. Convergent evidence from neuroimaging studies further supported a motor system dysfunction in SZ. Functional MRI (fMRI) studies, for instance, have revealed hyperconnectivity in the motor and somatosensory cortices of SZ patients [51]. Similarly, a magnetoencephalography (MEG) study found altered β-band oscillations in the pre-supplementary motor area and left motor cortex in SZ patients [52]. Furthermore, numerous studies have reported abnormalities in transcallosal motor inhibition in SZ [53]. However, it is important to note that some research has suggested that these inhibitory deficits may stem from dysfunctional cortical inhibitory mechanisms rather than primary CC pathway dysfunction [54, 55]. Consequently, future investigations should prioritize establishing direct relationships between these observed functional differences and structural abnormalities within the CC.

Between the ages of 11 and 15 years, CC exhibits faster growth rates in the isthmus and splenium, whereas changes in the rostrum and genu are nearly absent [56]; this may explain why abnormalities occur in the isthmus and splenium. The isthmus and splenium connect to the temporoparietal cortex, which is involved in spatial association and language functions, and to the occipital cortex, which is responsible for visual processing. Abnormalities in these brain regions have been reported in SZ [57, 58, 59], potentially playing a crucial role in the occurrence of hallucinations in patients [60, 61]. The efficacy of corpus callosotomy in ameliorating auditory and visual hallucinations in drug-resistant SZ further underscores the critical involvement of CC abnormalities in the generation of these hallucinatory symptoms in the disorder [62]. Recent findings have revealed that the primary fibers passing through the splenium link the precuneus on both sides [46]. This posteromedial parietal area is crucial for the default mode network [63] and is associated with self-perception and self-awareness [64]. Therefore, alterations in the CC splenium aligned with extensive studies that demonstrated how structural [65, 66, 67] and functional [68, 69] issues within the precuneus contribute to the underlying mechanisms of SZ. Additionally, some investigations have pointed to hippocampal connections that form a substantial portion of the ventral splenium [70, 71]. Therefore, the observed changes in splenium thickness in FES patients might be associated with the well-documented structural [72, 73], functional [74], and genetic [75, 76] abnormalities within the hippocampus in SZ.

Our PLSC analysis indicated that in older patients, greater thickness in the CC rostral body and splenium was associated with more severe affective symptoms, notably depression and anxiety. The sensitivity analysis indicated that this correlation remained robust after controlling for clinical characteristics. This finding aligned with evidence positing that CC abnormalities disrupt typical interhemispheric communication, potentially leading to the aberrant lateralization of emotional processing [77]. Given that emotional expression is predominantly governed by the right hemisphere (as evidenced by more intense left-sided facial expression [78]) and that right hemisphere lesions increase susceptibility to affective disorders [79], an enlarged CC may be associated with pathological interference with the normal hemispheric specialization required for the recognition, regulation, and expression of emotions. While our findings suggested a relationship between CC thickness and emotional dysfunction in older patients with FES, longitudinal research is needed to determine if this structural feature could serve as a reliable marker of disease progression. Furthermore, while these results highlighted the CC as a region of interest in emotional regulation, further research, especially interventional studies, will be valuable to determine whether this structure have practical utility for developing and refining interventions targeting emotional regulation. Our results further suggested a negative correlation between CC thickness and negative symptoms (specifically, stereotyped thinking). Previous studies have reported a similar inverse relationship between CC area and negative symptom scores in individuals with FES, despite no significant differences in CC area between patients and controls [80]. Findings from a diffusion tensor imaging study suggested that the fractional anisotropy value within the splenium of the CC is associated with negative symptoms in individuals diagnosed with SZ [81]. However, other investigations have not identified a significant relationship between CC structural abnormalities and negative symptom severity [82, 83]. Further investigation is crucial to clarify the specific role of CC in the manifestation of negative symptoms. The lack of a significant correlation between CC thickness and clinical symptoms in younger FES patients is likely attributable to ongoing white matter maturation during adolescence and early adulthood [33]. Within this neurodevelopmental phase, normative maturational processes overlap with disease-related pathological changes, potentially obscuring the expected relationship between CC abnormalities and clinical presentation. In contrast, among older individuals, after major neurodevelopmental trajectories have concluded, observed CC morphological alterations more directly reflect the pathophysiological impact of the disease itself.

### Limitations

This study has several limitations. First, the cross-sectional design precludes any conclusions regarding the temporal progression or stability of the observed structural brain differences. Second, potential confounding variables such as race, genetics, and significant differences in EDUY could affect CC thickness. Although EDUY was included as a covariate, this substantial difference may reflect broader population disparities, including socioeconomic status, which can also influence brain structure and FES pathology, thus precluding complete exclusion of their influence on our findings. Third, the current method for modeling CC thickness, using parallel non-overlapping streamlines, offers fine-grained detail along the sagittal plane but provides a narrow field of measurement, potentially overlooking crucial pathological features and limiting a comprehensive morphological profile. Furthermore, as noted by Owens-Walton et al. [84], a key limitation arises in regions of high curvature, such as the genu and splenium, where streamlines adopt curvilinear trajectories to preserve orthogonality with the superior and inferior margins of the structure. This complicates the interpretation of streamline lengths as direct measures of thickness.

## 5. Conclusions

In summary, our findings indicated less CC thickness in younger FES patients than in HCs, whereas older FES patients exhibited greater CC thickness. Furthermore, these differences were specific to particular subregions of the CC. Our findings may have elucidated the conflicting results observed in previous research and suggest that adolescent-onset SZ might represent a different form of the illness.

## Availability of Data and Materials

Imaging data supporting the findings of this study are available from the corresponding author upon reasonable request. All codes for analysis can be downloaded online from the following website: for CCSegThickness pipeline (https://github.com/chrisadamsonmcri/CCSegThickness), and for PLSC (https://github.com/danizoeller/myPLS/).

## References

[1] Insel TR (2010). Rethinking schizophrenia. *Nature*.

[2] GBD 2019 Mental Disorders Collaborators (2022). Global, regional, and national burden of 12 mental disorders in 204 countries and territories, 1990-2019: a systematic analysis for the Global Burden of Disease Study 2019. *The Lancet. Psychiatry*.

[3] Vaccaro J, Nili M, Xiang P, Nelson JK, Pack C, Thompson R (2025). Economic Burden Associated with Negative Symptoms Identified Through Natural Language Processing Among Patients with Schizophrenia in the United States. *Schizophrenia Bulletin*.

[4] Kelly S, Jahanshad N, Zalesky A, Kochunov P, Agartz I, Alloza C (2018). Widespread white matter microstructural differences in schizophrenia across 4322 individuals: results from the ENIGMA Schizophrenia DTI Working Group. *Molecular Psychiatry*.

[5] Zhuo C, Liu M, Wang L, Tian H, Tang J (2016). Diffusion Tensor MR Imaging Evaluation of Callosal Abnormalities in Schizophrenia: A Meta-Analysis. *PloS One*.

[6] Luders E, Thompson PM, Toga AW (2010). The development of the corpus callosum in the healthy human brain. *The Journal of Neuroscience: the Official Journal of the Society for Neuroscience*.

[7] Woodruff PW, McManus IC, David AS (1995). Meta-analysis of corpus callosum size in schizophrenia. *Journal of Neurology, Neurosurgery, and Psychiatry*.

[8] Arnone D, McIntosh AM, Tan GMY, Ebmeier KP (2008). Meta-analysis of magnetic resonance imaging studies of the corpus callosum in schizophrenia. *Schizophrenia Research*.

[9] Collinson SL, Gan SC, Woon PS, Kuswanto C, Sum MY, Yang GL (2014). Corpus callosum morphology in first-episode and chronic schizophrenia: combined magnetic resonance and diffusion tensor imaging study of Chinese Singaporean patients. *The British Journal of Psychiatry: the Journal of Mental Science*.

[10] Walterfang M, Wood AG, Reutens DC, Wood SJ, Chen J, Velakoulis D (2008). Morphology of the corpus callosum at different stages of schizophrenia: cross-sectional study in first-episode and chronic illness. *The British Journal of Psychiatry: the Journal of Mental Science*.

[11] Tepest R, Schwarzbach CJ, Krug B, Klosterkötter J, Ruhrmann S, Vogeley K (2013). Morphometry of structural disconnectivity indicators in subjects at risk and in age-matched patients with schizophrenia. *European Archives of Psychiatry and Clinical Neuroscience*.

[12] John JP, Shakeel MK, Jain S (2008). Corpus callosal area differences and gender dimorphism in neuroleptic-naïve, recent-onset schizophrenia and healthy control subjects. *Schizophrenia Research*.

[13] Coulon N, Godin O, Bulzacka E, Dubertret C, Mallet J, Fond G (2020). Early and very early-onset schizophrenia compared with adult-onset schizophrenia: French FACE-SZ database. *Brain and Behavior*.

[14] Hollis C (2000). Adult outcomes of child- and adolescent-onset schizophrenia: diagnostic stability and predictive validity. *The American Journal of Psychiatry*.

[15] Clemmensen L, Vernal DL, Steinhausen HC (2012). A systematic review of the long-term outcome of early onset schizophrenia. *BMC Psychiatry*.

[16] Witelson SF (1989). Hand and sex differences in the isthmus and genu of the human corpus callosum. A postmortem morphological study. *Brain: a Journal of Neurology*.

[17] Hofer S, Frahm J (2006). Topography of the human corpus callosum revisited–comprehensive fiber tractography using diffusion tensor magnetic resonance imaging. *NeuroImage*.

[18] Joshi SH, Narr KL, Philips OR, Nuechterlein KH, Asarnow RF, Toga AW (2013). Statistical shape analysis of the corpus callosum in Schizophrenia. *NeuroImage*.

[19] Adamson CL, Wood AG, Chen J, Barton S, Reutens DC, Pantelis C (2011). Thickness profile generation for the corpus callosum using Laplace’s equation. *Human Brain Mapping*.

[20] Annett M (2004). Hand preference observed in large healthy samples: classification, norms and interpretations of increased non-right-handedness by the right shift theory. *British Journal of Psychology (London, England: 1953)*.

[21] Chen J, Patil KR, Weis S, Sim K, Nickl-Jockschat T, Zhou J (2020). Neurobiological Divergence of the Positive and Negative Schizophrenia Subtypes Identified on a New Factor Structure of Psychopathology Using Non-negative Factorization: An International Machine Learning Study. *Biological Psychiatry*.

[22] Adamson C, Beare R, Walterfang M, Seal M (2014). Software pipeline for midsagittal corpus callosum thickness profile processing: automated segmentation, manual editor, thickness profile generator, group-wise statistical comparison and results display. *Neuroinformatics*.

[23] JOHNSON PO, FAY LC (1950). The Johnson-Neyman technique, its theory and application. *Psychometrika*.

[24] Miller JW, Stromeyer WR, Schwieterman MA (2013). Extensions of the Johnson-Neyman Technique to Linear Models With Curvilinear Effects: Derivations and Analytical Tools. *Multivariate Behavioral Research*.

[25] Bauer DJ, Curran PJ (2005). Probing Interactions in Fixed and Multilevel Regression: Inferential and Graphical Techniques. *Multivariate Behavioral Research*.

[26] Krishnan A, Williams LJ, McIntosh AR, Abdi H (2011). Partial Least Squares (PLS) methods for neuroimaging: a tutorial and review. *NeuroImage*.

[27] McIntosh AR, Lobaugh NJ (2004). Partial least squares analysis of neuroimaging data: applications and advances. *NeuroImage*.

[28] Walterfang M, Wood AG, Reutens DC, Wood SJ, Chen J, Velakoulis D (2009). Corpus callosum size and shape in first-episode affective and schizophrenia-spectrum psychosis. *Psychiatry Research*.

[29] Tao B, Xiao Y, Yang B, Zeng J, Zhang W, Hu N (2021). Morphological alterations of the corpus callosum in antipsychotic-naive first-episode schizophrenia before and 1-year after treatment. *Schizophrenia Research*.

[30] Nasrallah HA, Andreasen NC, Coffman JA, Olson SC, Dunn VD, Ehrhardt JC (1986). A controlled magnetic resonance imaging study of corpus callosum thickness in schizophrenia. *Biological Psychiatry*.

[31] Keshavan MS, Diwadkar VA, Harenski K, Rosenberg DR, Sweeney JA, Pettegrew JW (2002). Abnormalities of the corpus callosum in first episode, treatment naive schizophrenia. *Journal of Neurology, Neurosurgery, and Psychiatry*.

[32] Huang W, Chen M, Lyu G, Tang X (2021). A Deformation-Based Shape Study of the Corpus Callosum in First Episode Schizophrenia. *Frontiers in Psychiatry*.

[33] Lebel C, Deoni S (2018). The development of brain white matter microstructure. *NeuroImage*.

[34] Piekarski DJ, Colich NL, Ho TC (2023). The effects of puberty and sex on adolescent white matter development: A systematic review. *Developmental Cognitive Neuroscience*.

[35] Weinberger DR (1987). Implications of normal brain development for the pathogenesis of schizophrenia. *Archives of General Psychiatry*.

[36] Douaud G, Mackay C, Andersson J, James S, Quested D, Ray MK (2009). Schizophrenia delays and alters maturation of the brain in adolescence. *Brain: a Journal of Neurology*.

[37] Rosenthal R, Bigelow LB (1972). Quantitative brain measurements in chronic schizophrenia. *The British Journal of Psychiatry: the Journal of Mental Science*.

[38] Narr KL, Thompson PM, Sharma T, Moussai J, Cannestra AF, Toga AW (2000). Mapping morphology of the corpus callosum in schizophrenia. *Cerebral Cortex (New York, N.Y.: 1991)*.

[39] Aboitiz F, Scheibel AB, Fisher RS, Zaidel E (1992). Fiber composition of the human corpus callosum. *Brain Research*.

[40] David AS (1993). Callosal transfer in schizophrenia: too much or too little?. *Journal of Abnormal Psychology*.

[41] Nasrallah HA, McCalley-Whitters M, Bigelow LB, Rauscher FP (1983). A histological study of the corpus callosum in chronic schizophrenia. *Psychiatry Research*.

[42] Mamah D, Patel A, Chen S, Wang Y, Wang Q (2025). Diffusion basis spectrum imaging of white matter in schizophrenia and bipolar disorder. *Brain Imaging and Behavior*.

[43] Vanes LD, Mouchlianitis E, Wood TC, Shergill SS (2018). White matter changes in treatment refractory schizophrenia: Does cognitive control and myelination matter?. *NeuroImage. Clinical*.

[44] van der Knaap LJ, van der Ham IJM (2011). How does the corpus callosum mediate interhemispheric transfer? A review. *Behavioural Brain Research*.

[45] Xiong Y, Yang L, Wang C, Zhao C, Luo J, Wu D (2024). Cortical mapping of callosal connections in healthy young adults. *Human Brain Mapping*.

[46] Park HJ, Kim JJ, Lee SK, Seok JH, Chun J, Kim DI (2008). Corpus callosal connection mapping using cortical gray matter parcellation and DT-MRI. *Human Brain Mapping*.

[47] Zarei M, Johansen-Berg H, Smith S, Ciccarelli O, Thompson AJ, Matthews PM (2006). Functional anatomy of interhemispheric cortical connections in the human brain. *Journal of Anatomy*.

[48] Yüksek HH, Türkili S, Yüksek A, Ten B, Buturak Ş V (2025). Evaluation of Morphometric Findings of Corpus Callosum in Schizophrenia Patients with Magnetic Resonance Imaging and Comparison with Healthy Individuals. *Journal of Clinical Medicine*.

[49] Peretzke R, Neher PF, Brandt GA, Fritze S, Volkmer S, Daub J (2025). Deciphering white matter microstructural alterations in catatonia according to ICD-11: replication and machine learning analysis. *Molecular Psychiatry*.

[50] Walther S, Stegmayer K, Wilson JE, Heckers S (2019). Structure and neural mechanisms of catatonia. *The Lancet. Psychiatry*.

[51] Lányi O, Zahemszky D, Wenning AS, Engh MA, Molnár Z, Horváth AA (2025). Cerebello-Thalamo-Cortical Dysconnectivity in Schizophrenia Spectrum Disorders: A Resting-State Functional Magnetic Resonance Imaging Meta-Analysis. *Biological Psychiatry. Cognitive Neuroscience and Neuroimaging*.

[52] Han Y, Hua L, Xia Y, Sun H, Sheng J, Dai Z (2025). Neural correlates of behavioral control and impulsivity in first-episode schizophrenia: A MEG-Based beta oscillation analysis. *Journal of Psychiatric Research*.

[53] di Hou M, Santoro V, Biondi A, Shergill SS, Premoli I (2021). A systematic review of TMS and neurophysiological biometrics in patients with schizophrenia. *Journal of Psychiatry & Neuroscience: JPN*.

[54] Daskalakis ZJ, Christensen BK, Chen R, Fitzgerald PB, Zipursky RB, Kapur S (2002). Evidence for impaired cortical inhibition in schizophrenia using transcranial magnetic stimulation. *Archives of General Psychiatry*.

[55] Soubasi E, Chroni E, Gourzis P, Zisis A, Beratis S, Papathanasopoulos P (2010). Cortical motor neurophysiology of patients with schizophrenia: a study using transcranial magnetic stimulation. *Psychiatry Research*.

[56] Thompson PM, Giedd JN, Woods RP, MacDonald D, Evans AC, Toga AW (2000). Growth patterns in the developing brain detected by using continuum mechanical tensor maps. *Nature*.

[57] Tohid H, Faizan M, Faizan U (2015). Alterations of the occipital lobe in schizophrenia. *Neurosciences (Riyadh, Saudi Arabia)*.

[58] Teixeira S, Machado S, Velasques B, Sanfim A, Minc D, Peressutti C (2014). Integrative parietal cortex processes: neurological and psychiatric aspects. *Journal of the Neurological Sciences*.

[59] Schijven D, Postema MC, Fukunaga M, Matsumoto J, Miura K, de Zwarte SMC (2023). Large-scale analysis of structural brain asymmetries in schizophrenia via the ENIGMA consortium. *Proceedings of the National Academy of Sciences of the United States of America*.

[60] Cui Y, Liu B, Song M, Lipnicki DM, Li J, Xie S (2018). Auditory verbal hallucinations are related to cortical thinning in the left middle temporal gyrus of patients with schizophrenia. *Psychological Medicine*.

[61] Taylor M, Perera U (2015). NICE CG178 Psychosis and Schizophrenia in Adults: Treatment and Management - an evidence-based guideline?. *The British Journal of Psychiatry: the Journal of Mental Science*.

[62] Taghipour M, Ghaffarpasand F (2018). Corpus Callosotomy for Drug-Resistant Schizophrenia; Novel Treatment Based on Pathophysiology. *World Neurosurgery*.

[63] Raichle ME, MacLeod AM, Snyder AZ, Powers WJ, Gusnard DA, Shulman GL (2001). A default mode of brain function. *Proceedings of the National Academy of Sciences of the United States of America*.

[64] Cavanna AE (2007). The precuneus and consciousness. *CNS Spectrums*.

[65] Shapleske J, Rossell SL, Chitnis XA, Suckling J, Simmons A, Bullmore ET (2002). A computational morphometric MRI study of schizophrenia: effects of hallucinations. *Cerebral Cortex (New York, N.Y.: 1991)*.

[66] Schultz CC, Koch K, Wagner G, Roebel M, Nenadic I, Schachtzabel C (2010). Complex pattern of cortical thinning in schizophrenia: results from an automated surface based analysis of cortical thickness. *Psychiatry Research*.

[67] Venkatasubramanian G, Jayakumar PN, Reddy VV, Reddy US, Gangadhar BN, Keshavan MS (2010). Corpus callosum deficits in antipsychotic-naïve schizophrenia: evidence for neurodevelopmental pathogenesis. *Psychiatry Research*.

[68] Broome MR, Fusar-Poli P, Matthiasson P, Woolley JB, Valmaggia L, Johns LC (2010). Neural correlates of visuospatial working memory in the ‘at-risk mental state’. *Psychological Medicine*.

[69] Eisenberg DP, Sarpal D, Kohn PD, Meyer-Lindenberg A, Wint D, Kolachana B (2010). Catechol-o-methyltransferase valine(158)methionine genotype and resting regional cerebral blood flow in medication-free patients with schizophrenia. *Biological Psychiatry*.

[70] Colnat-Coulbois S, Mok K, Klein D, Pénicaud S, Tanriverdi T, Olivier A (2010). Tractography of the amygdala and hippocampus: anatomical study and application to selective amygdalohippocampectomy. *Journal of Neurosurgery*.

[71] Gloor P, Salanova V, Olivier A, Quesney LF (1993). The human dorsal hippocampal commissure. An anatomically identifiable and functional pathway. *Brain: a Journal of Neurology*.

[72] Roeske MJ, Konradi C, Heckers S, Lewis AS (2021). Hippocampal volume and hippocampal neuron density, number and size in schizophrenia: a systematic review and meta-analysis of postmortem studies. *Molecular Psychiatry*.

[73] Dong Q, Sheng Y, Zhu J, Li Z, Liu W, Liu J (2025). Schizophrenia Detection Based on Morphometry of Hippocampus and Amygdala. *IEEE Journal of Biomedical and Health Informatics*.

[74] Goghari VM, Sponheim SR, MacDonald AW (2010). The functional neuroanatomy of symptom dimensions in schizophrenia: a qualitative and quantitative review of a persistent question. *Neuroscience and Biobehavioral Reviews*.

[75] Weinberger DR (1999). Cell biology of the hippocampal formation in schizophrenia. *Biological Psychiatry*.

[76] Szeszko PR, Lipsky R, Mentschel C, Robinson D, Gunduz-Bruce H, Sevy S (2005). Brain-derived neurotrophic factor val66met polymorphism and volume of the hippocampal formation. *Molecular Psychiatry*.

[77] Wu JC, Buchsbaum MS, Johnson JC, Hershey TG, Wagner EA, Teng C (1993). Magnetic resonance and positron emission tomography imaging of the corpus callosum: size, shape and metabolic rate in unipolar depression. *Journal of Affective Disorders*.

[78] Sackeim HA, Gur RC, Saucy MC (1978). Emotions are expressed more intensely on the left side of the face. *Science (New York, N.Y.)*.

[79] Lishman WA (1968). Brain damage in relation to psychiatric disability after head injury. *The British Journal of Psychiatry: the Journal of Mental Science*.

[80] Takahashi M, Matsui M, Nakashima M, Takahashi T, Suzuki M (2017). Callosal size in first-episode schizophrenia patients with illness duration of less than one year: A cross-sectional MRI study. *Asian Journal of Psychiatry*.

[81] Arnedo J, Mamah D, Baranger DA, Harms MP, Barch DM, Svrakic DM (2015). Decomposition of brain diffusion imaging data uncovers latent schizophrenias with distinct patterns of white matter anisotropy. *NeuroImage*.

[82] Podwalski P, Tyburski E, Szczygieł K, Waszczuk K, Rek-Owodziń K, Mak M (2021). White Matter Integrity of the Corpus Callosum and Psychopathological Dimensions in Deficit and Non-Deficit Schizophrenia Patients. *Journal of Clinical Medicine*.

[83] Ahmadvand A, Shahidi SB, Talari H, Ghoreishi FS, Mousavi GA (2017). Morphology of the corpus callosum and schizophrenia: A case-control study in Kashan, Iran. *Electronic Physician*.

[84] Owens-Walton C, Adamson C, Walterfang M, Hall S, van Westen D, Hansson O (2022). Midsagittal corpus callosal thickness and cognitive impairment in Parkinson’s disease. *The European Journal of Neuroscience*.

